# 2D and 3D photonic crystal materials for photocatalysis and electrochemical energy storage and conversion

**DOI:** 10.1080/14686996.2016.1226121

**Published:** 2016-09-16

**Authors:** Gillian Collins, Eileen Armstrong, David McNulty, Sally O’Hanlon, Hugh Geaney, Colm O’Dwyer

**Affiliations:** ^a^ Department of Chemistry, University College Cork, Cork, Ireland; ^b^ Department of Life Science, Institute of Technology, Sligo, Ireland; ^c^ Micro-Nano Systems Centre, Tyndall National Institute, Cork, Ireland

**Keywords:** Photonic crystal, inverse opal, photoelectrochemistry, Li-ion battery, energy storage, energy conversion, catalysis, 50 Energy Materials, 105 Low-Dimension (1D/2D) materials, 204 Optics / Optical applications, 205 Catalyst / Photocatalyst / Photosynthesis, 206 Energy conversion / transport / storage / recovery, 207 Fuel cells / Batteries / Super capacitors

## Abstract

This perspective reviews recent advances in inverse opal structures, how they have been developed, studied and applied as catalysts, catalyst support materials, as electrode materials for batteries, water splitting applications, solar-to-fuel conversion and electrochromics, and finally as photonic photocatalysts and photoelectrocatalysts. Throughout, we detail some of the salient optical characteristics that underpin recent results and form the basis for light-matter interactions that span electrochemical energy conversion systems as well as photocatalytic systems. Strategies for using 2D as well as 3D structures, ordered macroporous materials such as inverse opals are summarized and recent work on plasmonic–photonic coupling in metal nanoparticle-infiltrated wide band gap inverse opals for enhanced photoelectrochemistry are provided.

## Graphical abstract

**Figure:**
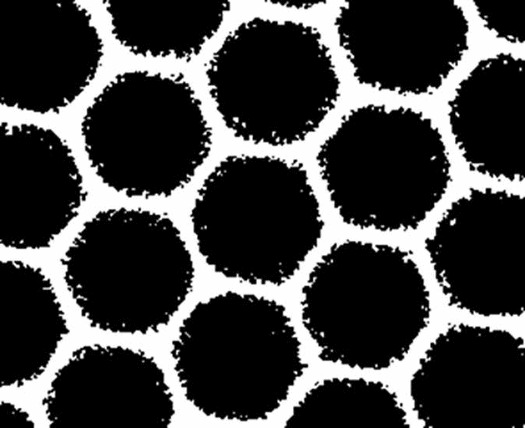

## 1. Introduction

Societal needs have driven the requirement for energy storage and conversion technologies that are cheap, stable, efficient and adaptable to a range of technology types (from personal devices to electric vehicles, to grid scale storage and conversion) using sources that are not limited. Energy conversion systems need energy storage systems to work in tandem. At the core of this necessary synergy are the materials responsible for storing and converting one form of energy to a combustible fuel, or from one form of energy to another, i.e. materials for battery electrodes, photoelectrocatalysis, hydrogen fuel production, solar cells, etc. Photocatalytic reactions are important for environmental remediation of persistent pollutants, disinfection, and energy production by water splitting. Batteries require material with structure and composition that are stable, provide high capacities over thousands of cycles, with faster charge rates, high energy density and conformability to several small and large technological systems, also a set of requirements for any good material and overall cell chemistry. These requirements have been reviewed in detail many times elsewhere.[1–6]

The field of photonic bandgap (PBG) engineering has matured in the last 30 years or so, from initial findings in enhancing light output and directionality for light emitting devices,[7,8] to photonic crystal (PhC) waveguiding in optics and optoelectronics,[9,10] to nanochemistry approaches to identifying the nature of light–matter interactions and how to control them [11] and recently, as metamaterials.[12] This was made possible by parallel advancement in creating these 3D structured materials using vacuum-based methods for high uniformity, to ever-improving colloidal and other methods to form opals and inverse opal (IO) materials. Synthesis aspects of inverse opals have been subject to excellent reviews and will not be considered here.[13] These photonic crystals are composite materials with a periodically varying refractive index, resulting in a band structure for photons that is conceptually similar to the electronic band structure in semiconductors – the correspondence lies in electron movement in an atomic lattice vs. photons moving through an optical lattice.[7,8] From the contrast in dielectric constant of the opal or inverse opal and surrounding medium, and the subsequent refractive index contrast within the photonic crystal, light can be scattered and diffracted causing destructive interference of scattered light waves in all directions – this provides a band of forbidden frequencies. Light cannot propagate within this PBG region, and the region’s spectral width is tuned by varying the refractive index contrast.[14] An incomplete PBG or pseudogap, sometimes referred to as a stop-band, prevents light propagation in some directions.

Opal colloidal crystals are typically made from equally sized polymeric or silica spheres assembled into a close-packed arrangement on a substrate, and they can be formed in 2D or 3D arrangement of sphere that exhibit characteristics very similar to natural opal or synthetic photonic crystals.[15,16] Inverse opals, by definition, comprise a solid architecture of material that matches the void space between the spheres of an opal. IOs are three-dimensionally ordered macroporous materials obtained by infiltration of an opal structure with a material with subsequent removal of the spheres (opal).[17] This leaves a structure identical to the void space of the parent opal and with a sufficiently high refractive index; IOs can exhibit a complete PBG. This optical characteristic has been applied to numerous applications ranging from energy storage and catalysis, to sensing [18] and optics.[19–21]

The need to develop smaller power sources was driven by requirements for so-called on-board microbattery power modules for microscale devices, and researchers are developing new materials to boost performance particularly for high energy density demands such as electric vehicles (EVs).[22] More recently, consideration for solid state batteries, thin film batteries and low volume cells that can operate under flexure or while stretched has been led by the hopes of developing smart textiles and flexible electronics where the rechargeable power source is integrated seamlessly with the form factor of the device or product.[5,23–26] These technological advancements have led to the search for 3D structured microbatteries, flexible and stretchable electrochemical energy storage and conversion technologies.[27,28] Insufficient power from 2D battery configurations, including the standard slurry mixture approach to cathode materials, was an open call for the development and improvement of 3D micro- or nanobatteries using cheap and light micro/nano fabrication materials and techniques.[29] At the core of these developments are the advancement in the type of materials, where composition, crystallinity, shape and chemical potential define the cycle life, capacity, voltage and energy density, etc.[2–4,30–37] Standard deposition of electrodes onto nanostructured current collectors has been proposed as an efficient and feasible route for the fabrication of 3D batteries or thin film battery electrodes with enhanced ambipolar (electronic and ionic) conductivity to enhance charge or discharge rates as required for fast charging consumer devices, or higher power delivery.[38] As outlined in [19,39], imposing a 3D or 2D structure on a battery material provides researchers with the opportunity to investigate the electrochemical response of the material structure, composition and crystallinity on the kinetics, cycle life and overall behaviour of newly developed materials options. We provide some details on these concepts here.

In the field of photoelectrochemical (PEC) water splitting, one of the best ways for solar to fuel production is the direct conversion of solar energy to hydrogen, effectively storing solar energy as a combustible gas. To boost efficiency in this process (so that it occurs at a very low voltage), single- or multi-component photoelectrode materials are being developed with very high electrochemical activity across the entire solar spectrum (not just the UV where most useful metal oxides absorb) while also maintaining long-term stability of the overall photoelectrode. The stability has been addressed somewhat in recent studies involving atomic layer processing of TiO_2_ layers with co-catalysts to give thousands of hours of continuous operation. Plasmonic metal nano-structures are becoming a material structure of choice for photoactive materials. They are highly stable and provide tunability in the light matter interactions that are useful for absorption, activity, catalysis, efficiency, etc. Properties such as surface plasmon resonance (SPR) of metal nanoparticles (NPs), absorption at the band edge of the support semiconducting materials, angle dependent or enhanced light capture by using PBG materials in energy regions where typically the absorption of the material itself is quite poor, plasmon-photon coupling scenarios, and tuning of the slow photon effect to match SPR resonances and thus charge injection, are all being assessed for PEC systems – we detail some of these advances in the reference cited below.

Here, we survey properties of 2D and 3D inverse opal or photonic crystal materials and examples of their development for use as catalysts and in several forms of photocatalytic systems, solar to fuel conversion and electrochromics, and as plasmonic–photonic photocatalysts. Finally, we examine characteristics of 2D and 3D ordered material form as structured electrode material for batteries and possibilities for optical analysis of charge-discharge and electrochemical effects that correlate materials structural and interfacial reactions to battery material response.

## 2. Inverse opals as catalysts and catalyst supports

Inverse opals possess several structural features making them ideal catalyst materials, such as high surface area and tunable macroporosity, stability and resistance to high-temperature coarsening. The co-existence of the interconnected macroporous and mesoporous structure enables favourable accessibility of the reactants to the active sites and enhanced catalytic activity is a general feature of the IO architecture, which has been demonstrated across a range of catalytic reactions where IOs can be used as catalysts and/or catalyst support materials. Umeda et al*.* [40] were one of the first to study the influence of the support material architecture on catalytic behaviour using ceria-zirconia (Ce_1−x_Zr_x_O_2_) IOs for propane oxidation. Using a non-templated ceria-zirconia powder as a control, they demonstrated the inverse opal catalyst displayed significantly higher catalytic activity. Grinding the IO to remove long range order decreased activity because the pulverized structure increased tortuosity for gas transport throughout the material. The IO version of the catalyst demonstrated excellent stability after continuous operation at 550 °C. Their inherent ordered macroporous structure facilitates mass transport due to reduced tortuosity, particularly beneficial for liquid phase reactions as demonstrated by carbon-coated TiO_2_ IO for methylene blue degradation. A comparison with mesoporous TiO_2_ structure showed the IO TiO_2_ structure performed better as the solution viscosity increased due to the low-tortuosity macropore network.[41]

**Figure 1.**
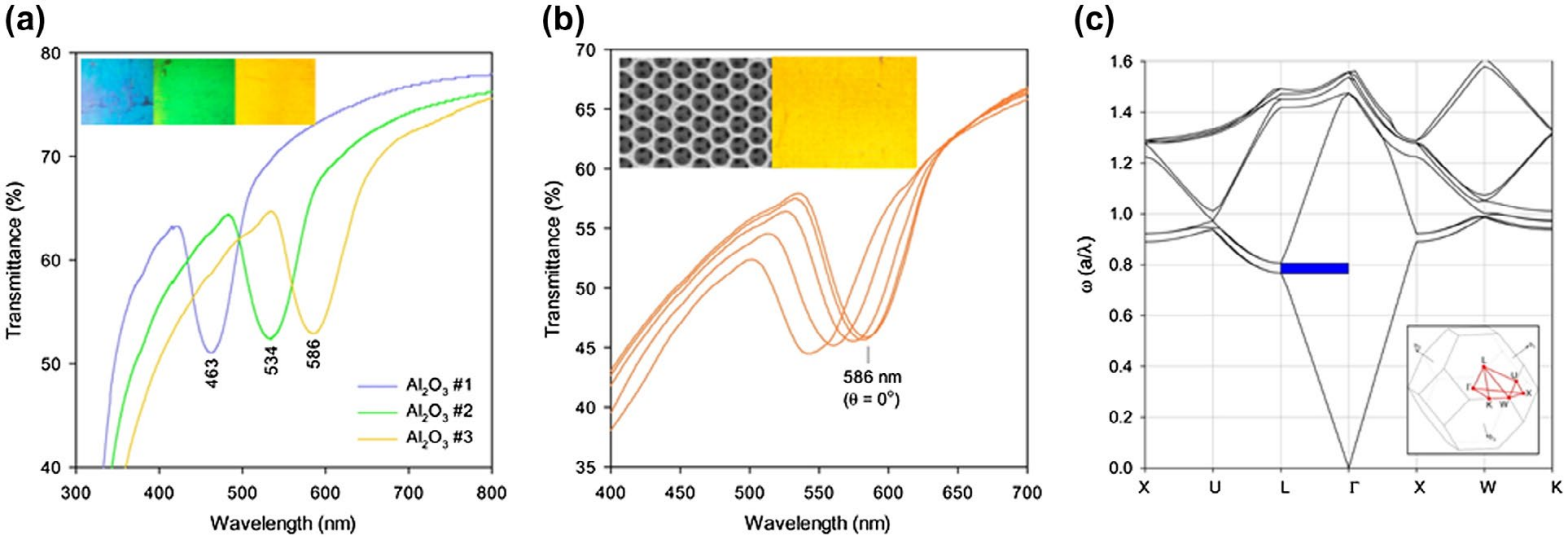
(a) UV-vis transmittance spectra for γ-Al_2_O_3_ inverse opal thin films in air showing photonic bandgaps along the [111] direction. Inset shows digital photographs of the γ-Al_2_O_3_ inverse opal thin films in air, illuminated and viewed along the [111] direction. The γ-Al_2_O_3_ inverse opals #1, #2 and #3 are inverse replicas of PMMA opals with defined sphere sizes (see [42]). (b) Consistent blue-shift of the UV-vis transmission suppression from the IO at incident angles of 0−20° with respect to the [111] direction. (c) Photonic band gap structure diagram for a γ-Al_2_O_3_ inverse opal showing a pseudo photonic bandgap between the second and third bands along the *L* → Γ direction (i.e. [111] direction). The inset shows the near spherical first Brillouin zone of a face centred cubic (fcc) lattice. Reproduced from [42] with permission from the American Chemical Society, 2015.

Waterhouse and co-workers [42] prepared high quality γ-Al_2_O_3_ IOs through top-down methods, as detailed in Figure 1. Importantly, the authors distinguished the dimensions of the IOs compared to the parent opal colloidal crystal template (sphere size) and confirmed a consistent variation in photonic band gap predicted by the periodicity and refractive index contrast of the resulting Al_2_O_3_. Onset temperatures for δ-Al_2_O_3_ → θ-Al_2_O_3_ → α-Al_2_O_3_ polymorphic transitions were found at ~50−100 °C higher for Al_2_O_3_ inverse opals compared to a sol−gel alumina nanopowder, indicating that the inverse opal architecture imparts sintering resistance, thus maintaining its structure and optical properties, which is particularly beneficial for high-temperature catalytic applications and for crystallizing important phases of the material without degradation of the 3D structure. They also demonstrated that Au/γ-Al_2_O_3_ catalysts synthesized using γ-Al_2_O_3_ inverse opal supports demonstrated excellent activity for carbon monoxide (CO) oxidation, whereby 69% CO conversion was possible at a low temperature of 20 °C with almost fully efficient conversion at 150 °C. Recently, integrating plasmonic NPs into IOs is becoming an important area of development and this is discussed in detailed in Section 3.2.

Ce_1−x_Zr_x_O_2_ is a commonly used support material for noble metal NPs in automotive catalysts designed for oxidation and reduction of combustion by-products, similar to catalytic converters. The low oxidation–reduction potential of the Ce^4+/^Ce^3+^ redox couple (1.61 eV) results in high mobility of lattice oxygen under reducing conditions and formation of oxygen vacancies under oxidizing conditions. Au [43] and Pt [44] supported NPs displayed enhanced catalytic performance in soot combustion attributed to structural effects associated with the 3D ordered macroporous (3DOM) architecture, improved the contact efficiency between the catalyst and soot, and synergistic metal-support interactions, where the support serves as an oxygen reservoir for the oxidation reaction. 3DOM IO La_1−x_KCo_3_ based on a perovskite-type oxide catalyst was also found to be a highly active metal-free catalyst for soot combustion.[44] Alumina is also a widely used catalytic support across a variety of applications including automotive and petro-refinery catalysts, and Fischer-Tropsch reactions. The advantages of using an IO architecture for the immobilization of catalytic nanoparticles has been demonstrated across a range of reactions such as Pd-γ-Al_2_O_3_ for catalytic methane combustion,[45] Pt-Rh-Al_2_O_3_ [46] and Au-Al_2_O_3_ [42] for CO oxidation and CoMo-Al_2_O_3_ for hydrodesulfurization of dibenzothiophene.[47]

**Figure 2.**
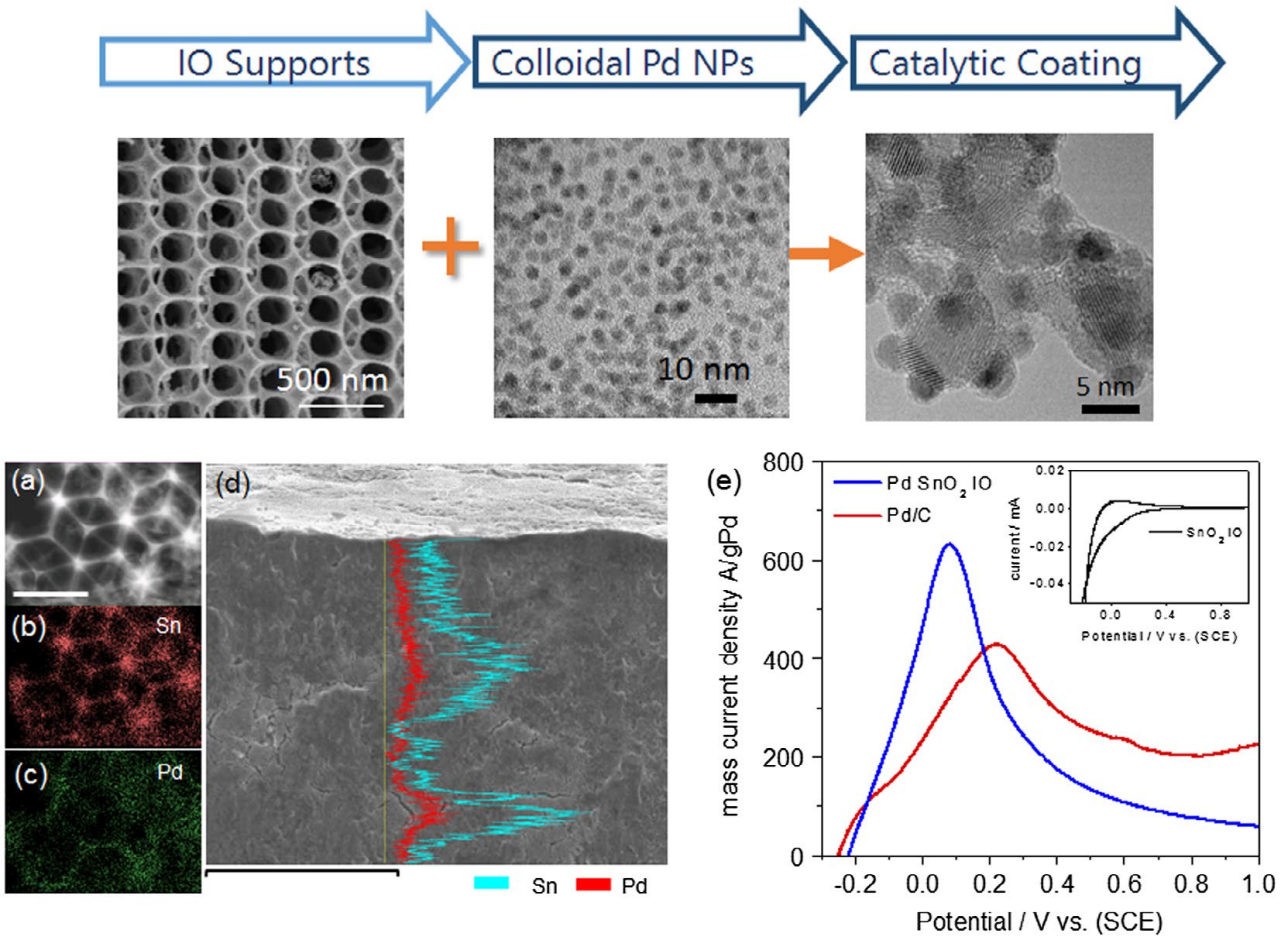
(Top) SnO_2_ IOs formed using double infiltration of a Sn(IV) acetate, calcined at 0.5 °C min. As-synthesized OA-capped Pd NPs are immobilized onto SnO_2_ IOs. (a) Scanning transmission electron microscopy (STEM) image of Pd deposited on SnO_2_ IO at its (111) orientation. Scale bar = 500 nm. Corresponding elemental maps are shown for (b) Sn and (c) Pd. (d) Cross sectional scanning electron microscopy (SEM) image of SnO_2_−Pd IOs and overlaid energy dispersive X-ray (EDX) spectroscopy line scans for Sn and Pd. Scale bar in part d is 100 μm. (e) Linear sweep voltammogram of formic acid oxidation using Pd SnO_2_ IOs. Reproduced from [48] with permission from the American Chemical Society, 2013.

Inverse opal catalyst supports of Pd NPs have also been synthesized with high NP loading and a well-defined interaction with the host support. Collins et al. [48] developed SnO_2_ IOs (see Figure 2) with hierarchical length scales or porosity and structure from nm to cm, with wall thickness of the IO (comprising SnO_2_ nanocrystals) tunable by the number in infiltrations used during the templating step. Attaching metal NPs to any IO is an important step when metal-semiconductor interactions are required from the device or material. For electrical and optical reasons, efficient electron transport and a well-defined Schottky barrier is necessary in photoelectrodes for example, while chemical stability of the NP adhered to the IO walls is crucial for stable behaviour during solution-based chemical catalysis. Here, weak ligand−metal interactions and strong metal–oxide interactions ensure good Pd NP docking throughout the IO, even down to depths of several mm thickness. These IO catalyst supports show superior catalytic performance for liquid phase chemical synthesis via Suzuki coupling reactions and allow easy catalyst removal after the reaction. Higher mass electrocatalytic activity is also demonstrated for formic acid oxidation compared to commercial Pd/C catalysts, taking advantage of good wetting of the Pd/SnO_2_ IO catalytically active sites.

## 3. Inverse opals as photocatalysts and photoelectrochemical cells

Photocatalysts and photoelectrochemical cells are the corner stone for applications including solar fuel generation, pollutant remediation and photocatalytic synthetic reactions.[49] IOs provide several structural features similar to photonic crystals and are considered a useful architecture for photoelectrochemical (PEC) applications. Nano-structuring has been extensively studied, reducing bulk recombination by shortening the diffusion length for charge carriers. TaON [50] and BiO_4_ IOs [51] show improved charge migration as the interconnected periodic macroporous architectures provide long-range-ordered paths for electron transport throughout the electrode. Furthermore, the facile precursor infiltration preparation method enabled easy partial substitution of V^5+^ with of Mo^6+^, resulting in higher charge carrier concentration for Mo-doped BiVO_4_ IOs.[52] Cadmium chalcogenide nanocrystals typically display low PEC efficiency, but incorporating CdS into an IO structure resulted in the unprecedented PEC activity and stability. CdS IO displayed the highest H_2_ production rate reported for cadmium chalcogenide nanocrystals with more than three times greater photocurrent density, improved light absorption and charge separation compared and efficiency of CdS thin films.[53] The origin of the enhancement was attributed to the IO structure overcoming the intrinsically slow hole transfer kinetics typical of CdS photoanodes. Zhou et al. [51] conducted an interesting study investigating how the dual porosity influenced charge migration in BiVO_4_ IOs for PEC water splitting and showed a strong dependence on the relative pore size. The porosity of IOs are characterized by the diameter of the macropore, determined by the size of the original sphere template, and the diameter of the pore between the neighbouring macropores, determined by the interconnected points between the spheres. Smaller macropore diameter facilitates surface migration but impedes the bulk charge migration while a smaller pore diameter between the neighbouring macropores is beneficial for charge migration in the bulk and on the surface.

To overcome the shortcomings of an individual semiconductor for charge generation, electron–hole pair separation and band gap, the use of heterojunctions and composite materials are a common design for PEC catalysts. The versatile synthesis methodologies of IOs make them compatible with the synthesis of composite photoanodes of p-type and n-type materials. WO_3_/BiVO_4_ inverse opal photoanodes demonstrated a photocurrent that was ∼40 times higher than that of the pure inverse opal WO_3_ photoanodes.[54] This material had a core-shell structure, where the WO_3_ core was stated to have better electron transporting properties than the BiVO_4_ shell, the latter having a more favourable band gap energy for solar harvesting. The BiVO_4_ coated onto the WO_3_ skeleton leads to efficient charge generation and separation, thereby improving the photocurrent density remarkably. Similar enhancement mechanisms were observed for WO_3_ coated SnO_2_ IOs.[55] A α-Fe_2_O_3_ and graphene composite exhibited 1.4 × increased efficiency in water splitting relative to pristine α-Fe_2_O_3_.[56] The graphene interlayer can act as both an electron transfer layer and an electrolyte blocking barrier, which not only reduces the charge recombination at the substrate–electrolyte interface but also helps electron transportation from α-Fe_2_O_3_ to the photoanode.[57] Combining ZnO nanowires and TiO_2_ inverse opals showed enhanced photocurrent improvement in comparison to that of pure ZnO due to electronic and optical coupling.[58] SnO_2_ IOs surrounded by a 10–40 nm TiO_2_ shell enhanced photo current density by three orders of magnitude due to low charge-transfer resistance at the SnO_2_/TiO_2_ interface as well as benefiting from the smaller TiO_2_ bandgap.[59] Coridan and co-workers [60] used an innovative approach to introduced porosity into core-shell photoanodes by coating silicon microwires with WO_3_ IOs shells.

### 3.1. Photonic photocatalysts incorporating the slow photon effect

The unique optical properties of photonic crystals (PhCs) enables them to manipulate and confine light, providing new opportunities and strategies to improve efficiency in photocatalysts and photoelectrodes. Porous photonic crystal structures can reduce light loss due to reflection, as photons that enter the inverse opals undergo multiple scattering by the walls making them less likely to escape.[61] This confinement of light has been used in photonic crystal structures including IOs, and nanotube arrays. In some instances, PhCs confine light emitted from the material that it is constructed from, resonantly enhancing the light intensity of the output emission. Similarly, IOs in the form of PhCs also provide angle-dependent reflection or waveguiding, depending on the physical parameters of the IO structure (see [19,62–64] and related reviews for further details on the basic optical properties of PhCs and the slow photon effect in particular). More significantly, photons in inverse opals propagate with strongly reduced group velocity at the frequency edges of the photonic stop band. The group velocity is the velocity with which the envelope of a light wave propagates through a medium. At the edges of the PBG, light travelling with a strongly reduced group velocity gives rise to the slow photon effect enabling enhanced light–matter interaction. At the red edge or lower frequency end of the PBG, the incident light is localized on the dielectric material, for example TiO_2_. If the red edge of the TiO_2_ IO PBG was tuned to overlap with the electronic absorption band edge of TiO_2_, which lies in the UV (~370 nm), optical absorption is enhanced due to the longer photon lifetime in the photonic crystal. This in turn gives rise to increased generation of electron–hole pairs and higher photocatalytic activity, when used in electrochemical cells. The slow photon effect has gained much attention due to the great potential in enhancing light absorption and photocatalytic performance. Several reports of enhanced light absorption at the blue edge of the PBG have been reported.[65] A theoretical investigation into this phenomenon demonstrated that the accepted theory on light intensity confinement in high dielectric constant regions is flawed when applied to the IO structure due to the subwavelength size of the material architecture.[66] Computational studies demonstrated that blue edge photons can also experience enhanced absorption. The origin lies in loose confinement due to the dimension of the IO skeleton, i.e. while the intensity was concentrated in the voids, the blue edge photons tunnel across the IO structure experiencing resonantly enhanced intensity. This loose confinement results in significant overlap with the IO skeleton and so leads to absorption enhancement.[66]

Ozin and co-workers were the first to demonstrate the phenomenon of slow photons for enhanced catalyst in degradation of methyl orange (MO) using a TiO_2_ IO.[62,67] In a later study they investigated tuning the PBG in trans-cis photo-isomerization of azobenzene by functionalizing SiO_2_ IOs with the azobenzene molecules.[68] While TiO_2_ has been by far the most studied IO material,[69] a variety of oxides and multi-oxide compositions are emerging due to the compositional diversity attainable with the IO synthesis methodology. ZnO_2_ IOs have be used in the degradation of methyl orange, methylene blue (MB), rhodamine blue (RhB),[65,70] Fe_2_O_3_ IOs for crystal violet degradation,[71] nitrogen-fluorine co-doped TiO_2_ IOs for RhB,[72] and β-Ga_2_O_3_ IOs for degradation of several organic pollutants.[73]

**Figure 3.**
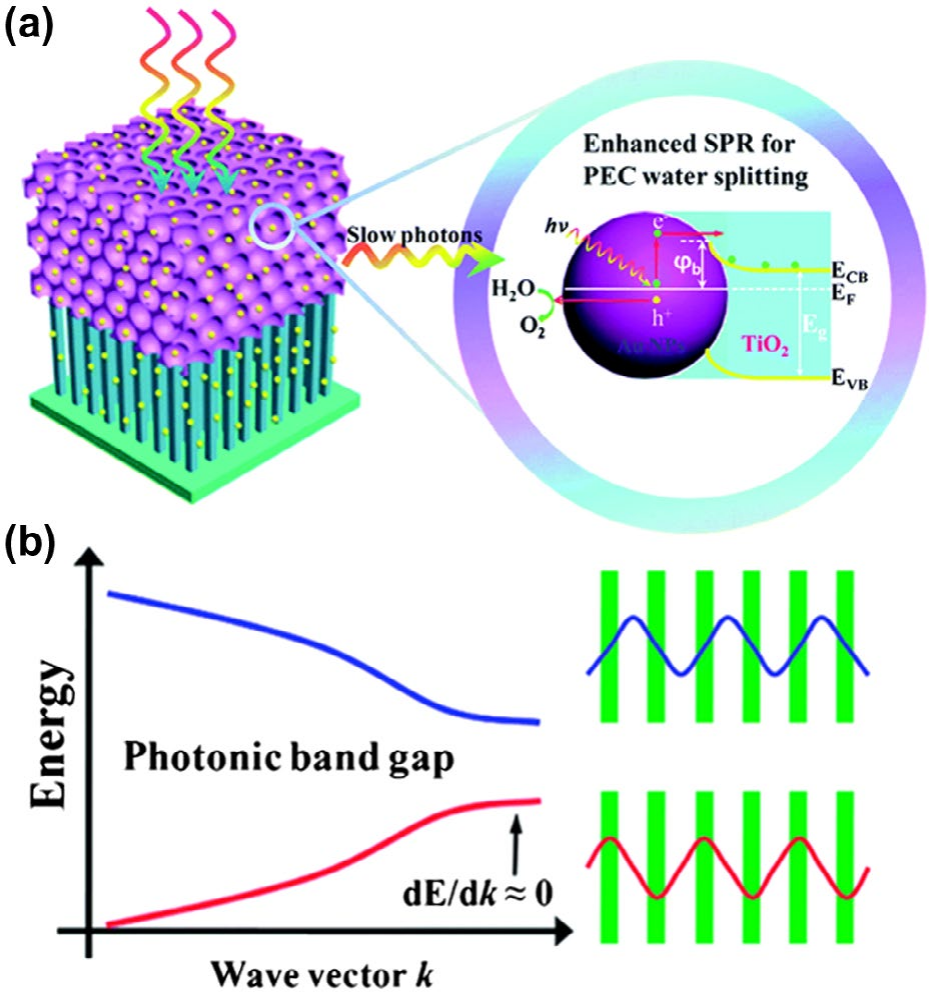
(a) Schematic diagram for the synergy of the slow photon effect of TiO_2_ PC and SPR enhanced PEC water splitting under light illumination. The left panel is the schematic drawing of Au/TiO_2_ NRPCs. Light transmission with a decreased group velocity in the TiO_2_ PC layer at the red edge of PGB (i.e. slow photon effect). The right panel is the energy diagram of the composite plasmonic photoanode and the mechanism of SPR PEC water splitting. (b) Schematic optical band structure of PCs and electric field distributions of light near the blue edge and red edge of the photonic band gap. The green part indicates the high-dielectric region relative low-dielectric interval region. Reproduced from [63] with permission from the Royal Society of Chemistry, 2014.

Many of these studies prove that matching the PBG of the 3DOM structures with the electronic band gap of photocatalysts results in a significant enhancement of photocatalytic activity under light irradiation. Dual enhancement effects were observed in ZnGa_2_O_4_ IOs for degradation of MO due to the IO architecture which facilitated mass transport in a highly ordered channel coupled with the slow photon effect.[74] Using wavelengths well below the PBG where the slow photon effect would not occur, strong light scattering effects were observed in enhancing photocatalytic decomposition of isopropanol Au-TiO_2_ IOs.[75] Zhang and co-workers [63] prepared Au NP decorated TiO_2_ IOs where the red edge of the PBG was tuned to overlap with the LSPR of the Au NPs, resulting in one of the of the highest reported photoconversion efficiencies for PEC water splitting using an Au-TiO_2_ system (0.17%). Figure 3 summarizes this result and highlights the basics of the slow photon effect schematically. The result was an elegant way of showing that the enhanced light capture by the inverse opal photonic crystal photogenerates *e*-*h* pairs, which are excited from the Au NPs to the conduction band of TiO_2_, with the Schottky barrier minimizing reverse carrier transport. The slow photon effect then enhances the surface plasmon resonance of ‘hot’ electrons added to the TiO_2_, which enhances the PEC efficiency greatly for water oxidation.

The slow photon effect has also been utilized for photoelectrochemical conversions. Tungsten trioxide (WO_3_) photoanodes are promising material for the conversion of solar energy into H_2_ due to their visible light response and good photochemical stability.[70] WO_3_ fabricated as a 3D photonic crystal photoanode displayed enhanced photon-to-electron conversion efficiency. Furthermore, when the red-edge of the photonic stop-band of WO_3_ IOs overlapped with the WO_3_ electronic absorption edge at *E*_g_ = 2.6–2.8 eV, a maximum of 100% increase in photocurrent intensity was observed under visible light irradiation (λ > 400 nm) for disordered IOs in comparison with a disordered porous WO_3_ photoanode. Introducing mesoporosity into the IO wall using polyethylene glycol as an organic surfactant can further increase specific surface area and enhanced PEC activity.[76]

### 3.2. Nanoparticle decorated inverse opals – plasmonic–photonic coupling in inverse opal catalysts

Another modern design strategy for photonic crystal photocatalysts is the incorporation of metal NPs onto semiconductor photocatalysts, effectively creating a Schottky junction in tandem with enhancing photoconduction of the semiconducting oxide by promotion of hot electrons from the adsorbed metal NP. This effect can be exploited in reverse – photoexcited *e*-*h* pairs that couple to metal NPs above a threshold related to the barrier height of the Schottky junction, and both effects are enhanced by fashioning the metal oxide into an IO opal that can resonantly enhance light capture or promote light absorption at certain wavelengths and certain angles. At a metal–semiconductor junction, it is well established that the barrier height limits electron flow from the semiconducting IO into the metal, unless the electron affinity of the semiconductor is close to the workfunction of the metal – then, the metal centres act as electron sinks.[77] The enhanced separation of photo-generated charge carriers (with the barrier height preventing recombination) leads to longer lifetimes and increased photocatalytic activity. Ozin and co-workers were also the first to demonstrate that the slow photon effect could be combined with a chemical enhancement effect in Pt NP-decorated TiO_2_ IOs.[78] They observed a fourfold increase in efficiency for photo-degradation of acid orange compared to the bare TiO_2_ IO. Interestingly, immobilizing the Pt onto disordered TiO_2_ only produced a 1.8 × increase in efficiency, indicating cooperative effects between slow photons and the extended *e*-*h* lifetime by the incorporation of Pt NPs.

Plasmonic metallic nanostructures have attracted considerable attention in photocatalytic and PEC areas due to their strong interactions with light through excitation of surface plasmon resonance (SPR). Incorporating plasmonic nanostructures into wide band gap semiconductors is an effective strategy to improve visible light response. The origins of plasmon-assisted photocatalytic processes has been attributed to several effects including local heating, plasmon-induced charged transfer, enhanced charge carrier separation and local electric field enhancement. As photonic crystals such as IOs enable tuning of light-matter interactions through nano-structuring of the support material in principle, photocatalytic performance can be enhanced through a combination of photonic and plasmonic components.[79,80] Synergetic effects of the photonic crystal stop bands and plasmonic absorption were observed in Au decorated BiVO_4_ IOs photoanodes for water splitting, with larger enhancements up to 700% observed at 520 nm due to plasmonic coupling.[81] The work showed that the photocurrent enhancement induced by the Au NPs was greater with the IO architecture compared with unstructured planar photoanodes and disordered IOs and this enhancement was attributed to the synergistic effect of photonic Bragg resonances and the plasmonic resonance of the Au NPs. However, for all these forms of NP-infiltrated IO systems the actual dispersion of NP throughout the IO at electrode level is often missing, particularly metallic NP aggregation within characteristics cracks in large area opals. In addition to PEC applications, performance enhancement using plasmonic NPs has been demonstrated in solution phase reactions such as Ag-TiO_2_ [82] and Ag-BiVO_4_ [83] IOs for MB degradation. Incorporation of Au NPs intoTiO_2_ IOs enhanced visible light degradation of 2,4-dichlorophenol which was ascribed to both Bragg diffraction and slow photon effects coupled with the LSPR of the NPs.[84]

**Figure 4.**
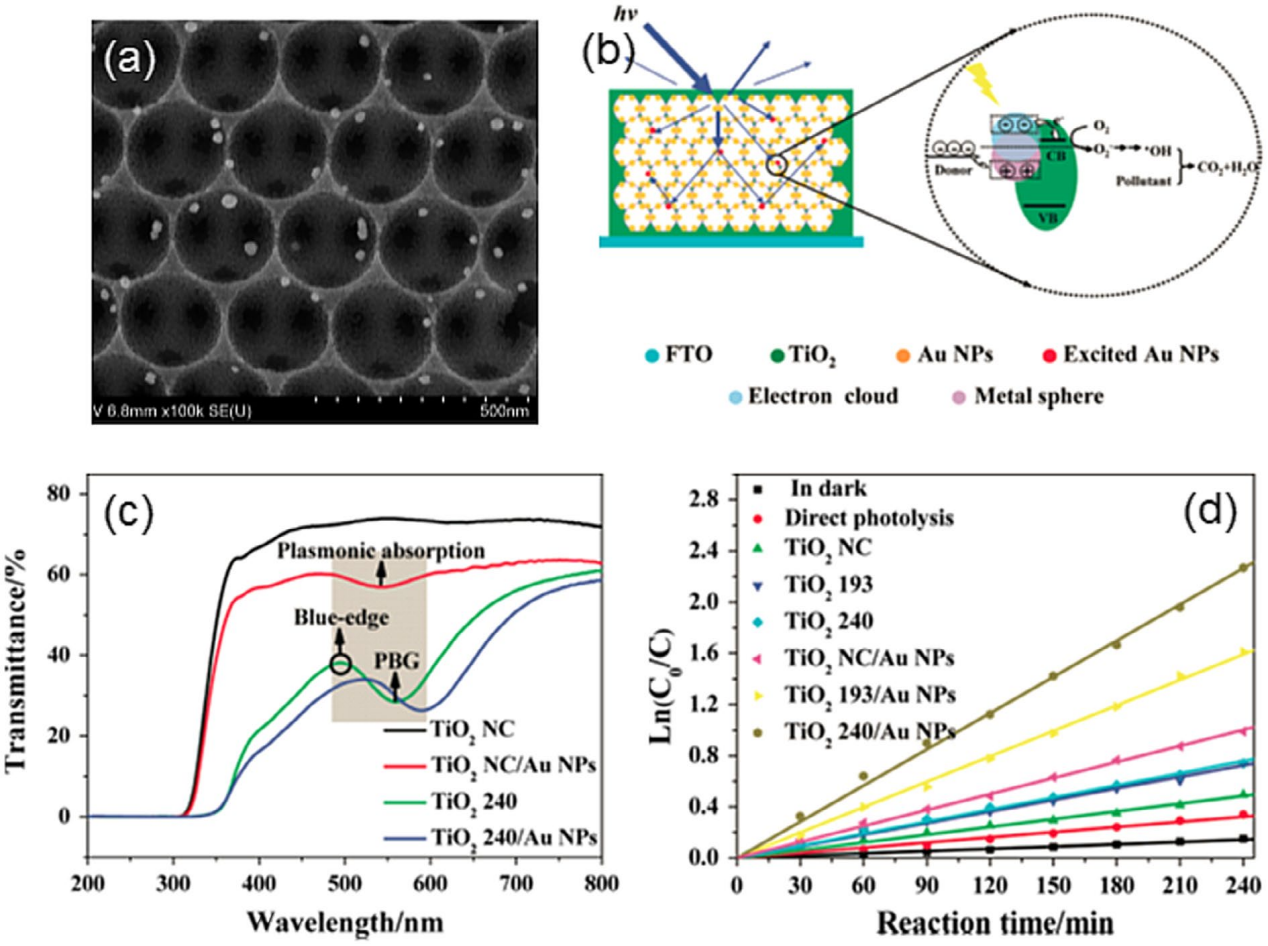
(a) SEM image of TiO_2_/Au NPs IO prepared with 240 nm PS spheres. (b) Schematic illustration of pollutants degradation mechanism using TiO_2_ PhC/Au NPs under λ > 420 nm irradiation. The blue lines represent the process of Bragg reflection, diffuse scattering and multiple internal scattering in TiO_2_ PhC. (c) Transmittance spectra of TiO_2_/Au NPs. The shaded region indicates the overlapping wavelengths between the photonic band gap of TiO_2_ and the plasmonic absorption by Au NPs, and the circle represents the blue-edge of the photonic band gap. The sphere size in the template was 240 nm. (d) The kinetics of 2,4-DCP degradation using various photocatalysts under visible light (λ > 420 nm) irradiation. Reproduced from [84] with permission from the Royal Society of Chemistry, 2012.

In Figure 4, the structure of the NP-infiltrated TiO_2_ IO and the modification of optical absorption across the band covered by the blue edge of the material’s absorption and the PBG by Au NPs, confirms the salient features of plasmon enhanced photoelectrochemical reactions aided by photonic band gap materials. One should also be cognisant of the fact that in these materials, ordered or not, considerable scattering does occur and some wavelengths are internally reflected, causing enhanced absorption that is not specifically due to an optical characteristic of the ordered (photonic crystal-like) nature of the host oxide per se. Additionally, these designs can benefit from enhanced light guiding at angles where the sunlight is lower in the sky, but any wavelength dependence must ensure that ballistic phonon transport or others form of light leakage do not reduced the degree of light–matter interaction that is critical to the operation of these forms of photoelectrodes.

A variety of NP materials including carbon quantum dots in BiVO_4_ [85] and CdS and CdSe in TiO_2_ [86,87] have also been used for photochemical H_2_ generation. The chemical diversity of the IO synthesis has allowed complex ZnO/Au/CdS sandwich structure IO, where synergistic effect between the plasmonic Au nanoparticles and CdS quantum dots improves the visible light harvesting.[88] Enhanced photocatalytic activity has also been achieved by matching the photonic band gap of TiO_2_ to the absorption peaks of dyes.[89]

In addition to photonic photocatalysts, i.e. where the active material is photoactive, photochemically inactive IO hosts can be used as support material for photocatalytic nanoparticles, as demonstrated by Mitchell et al. [90] They synthesized ZrO_2_ IO decorated with CdS NPs for photocatalytic hydrogen production from water. The absorption band of the CdS NPs partially overlaps with the blue edge of the photonic ZrO_2_ stop band giving rise to a nearly fivefold enhancement in H_2_ production compared to CdS deposited on a non-photonic support. This general strategy potentially allows any photoactive material to be incorporated into a photonic host material.

## 4. Electrochromism and 3D chromogenic materials

Electrochromic materials have been developed considerably over the last 20 years and have found application in smart windows, display devices, controlled reflectance mirrors, and even in thermal control devices for space shuttles. Good electrochromic materials require small amount of energy (low voltages) to drastically alter their coloration, but the best chromogenic materials keep a low power requirement to maintain that coloration state without bleaching or change. The bleached and coloured state in metal oxide electrodes can be switched by electric-field induced intercalation of ions into the lattice, much like a lithium or lithium-ion battery, but a primary difference is often the use of aqueous electrolytes.[91–93] Electroneutrality is maintained by charge transfer that involves both ions and electrons, and recent research advances are focused on enduring very high optical contrast between ON and OFF states, and faster rates of switching. While the optical contrast and optical saturation are a function of the absorption characteristics of the parent metal oxide, the rate of contrast change is an ion diffusion problem – a similar consideration to metal oxides for faster charging Li-ion battery electrodes for example. Conventional flat electrochromic films are thin films solids and of course, solid state diffusion limitations affect coloration rates and optical contrast – while resistivity and methods of growth affect electron conductivity in parallel.

**Figure 5.**
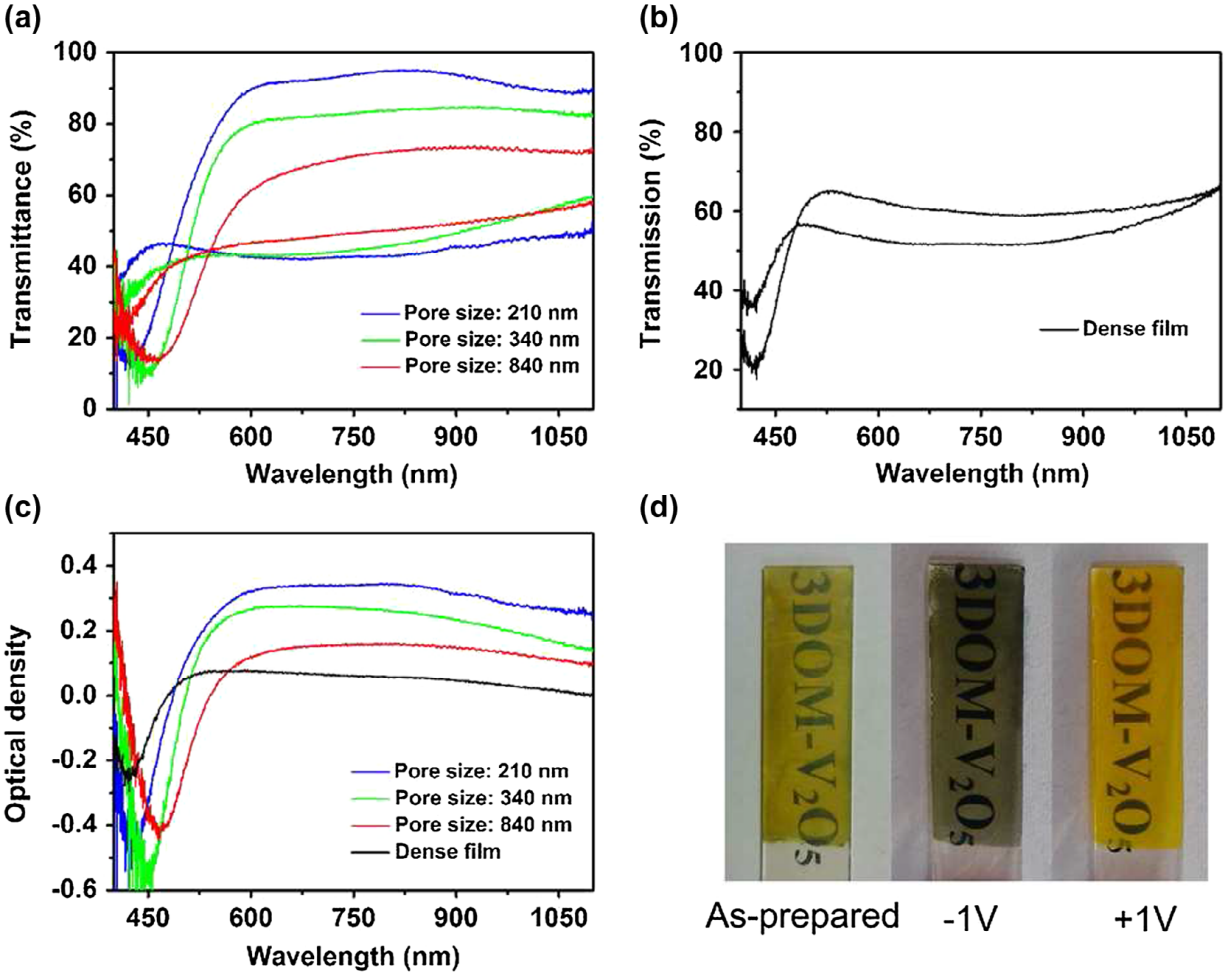
Transmittance contrast of (a) the 3DOM V_2_O_5_ films and (b) the dense V_2_O_5_ film; (c) optical density of both the 3DOM and the dense films, and (d) electrochromic digital photographs of a 3DOM V_2_O_5_ film with pore size of 210 nm, under different potentials. Reproduced from [94] with permission from the Royal Society of Chemistry, 2014.

Analogous to Li-ion batteries and pseudocapacitors too, electrochromic materials involving intercalation mode operation also benefit from 3D structures, electrolyte penetration to reduce diffusion constants, interconnected material networks to ensure good electrical conductivity, and modes of formation such as electrodeposition that ensure good adhesion to any current collector or substrate. Using V_2_O_5_ as a good chromogenic material, Li et al. [94] showed that cation diffusion constants can be significantly reduced in deposited IOs, thus improving electrochromic response (see Figure 5). Small pores lead to higher optical contrast and faster switching response. A high transmittance modulation of 50% at 650 nm and 47% at 900 nm with a fast response time of 1.7 s for coloration and 3.2 s for bleaching is possible from IOs with 210 nm voids, made possible by a high Li-ion diffusion coefficient of 3.78 × 10^−9^ cm^2^ s^–1^.

Electrochromic phenomena are similar to intercalation pseudocapacitance, without formal reactions between the host and cation in the latter case. In 3D structured materials, the benefits of the ordered porosity and conductivities for electrons and ions are expected to some degree. However, optical analysis of the PBG and reflectance characteristics also provide ways of assessing the optical conductivity variation by linking optical reflectance to cation insertion effects on materials structure, order, periodicity and associated PBG change. Deconvolution of material electrochromism under optical excitation vs. electrochemically induced colour changes from variation in periodicity, pore volume, material dielectric constant, etc., are critical for understanding the contribution of the material to electrochromism, from structural colour changes which are angle dependent.

## 5. Photonic crystal properties for energy storage

### 5.1. Inverse opals and 3D structured materials for battery electrodes

The first report of forming battery electrode materials using colloidal crystal templates was in 2002.[95] In that paper, Sakatomo et al. reported the synthesis of V_2_O_5_ with pore sizes ranging between 10 and 30 nm that delivered higher capacities at higher discharge rates because of improved charge transport. Since then, many other high surface area electrode designs have been prepared and tested including a copper pillar array,[96–98] aluminium nanorod array,[99] nickel network,[100] stainless steel mesh,[101] polymer scaffold,[102] and coaxial,[103] nanotube [104] and nanofoam [105] carbonaceous interpenetrating structures to form conductive pathways in battery cathodes and anodes – advancements in 3D structured materials and current collectors has shown what parameters can be modified in battery systems.[19,98,106–109] Limitations in performance can also come from battery material resistances and electrical disconnectivity during cycling, which is something of concern. To overcome this, the use of 3D structured current collectors was posited to keep ion and electron diffusion distances as small as possible; an influence that can be rationally tuned and tested with this design.[110,111]

**Figure 6.**
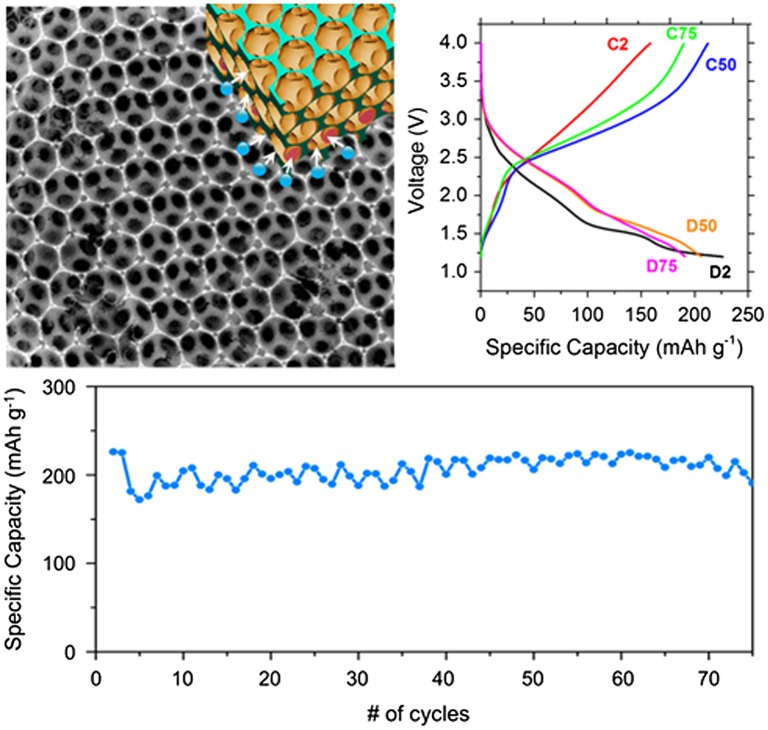
Electrodeposited V_2_O_5_ inverse opal structure amenable to electrolyte infiltration and short diffusion length for Li-ion during reversible insertion and removal as a Li-ion battery positive electrode. Such structures offer not only high capacities, but negligible initial capacity fade and efficient Coulombic charge-discharge cycling over 75 cycles. Reproduced from [112] with permission from the American Chemical Society, 2015.

Very recently, excellent work where the Ni current collector was structured using a similar approach [113] allowed for much better active material coverage of the current collector and charging rates of up to 1000 C, but with limited cycling capability. Elsewhere, the reader can see a comparison of electrode materials performance in Li-ion batteries linked to a stated modification of the overall electrode structure [19] and we refer the reader to Stein’s opinions on what makes inverse opal and 3D structured battery electrodes materials special.[114] Separately, inverse opal-structured electrodes demonstrated promising electrochemical behaviour, whereby the porous structure was soaked with electrolyte in a flooded cell (to ensure complete soakage and remove any limitations on Li diffusivity from air pockets, and mass transports of species).[112,115] Figure 6 shows the excellent cycling stability of these inverse opal thin film lithium batteries that was due to ensuring stable interfacial and IO network mechanical structure and electrical connectivity with the current collector since the IO was formed by electrodeposition.

**Figure 7.**
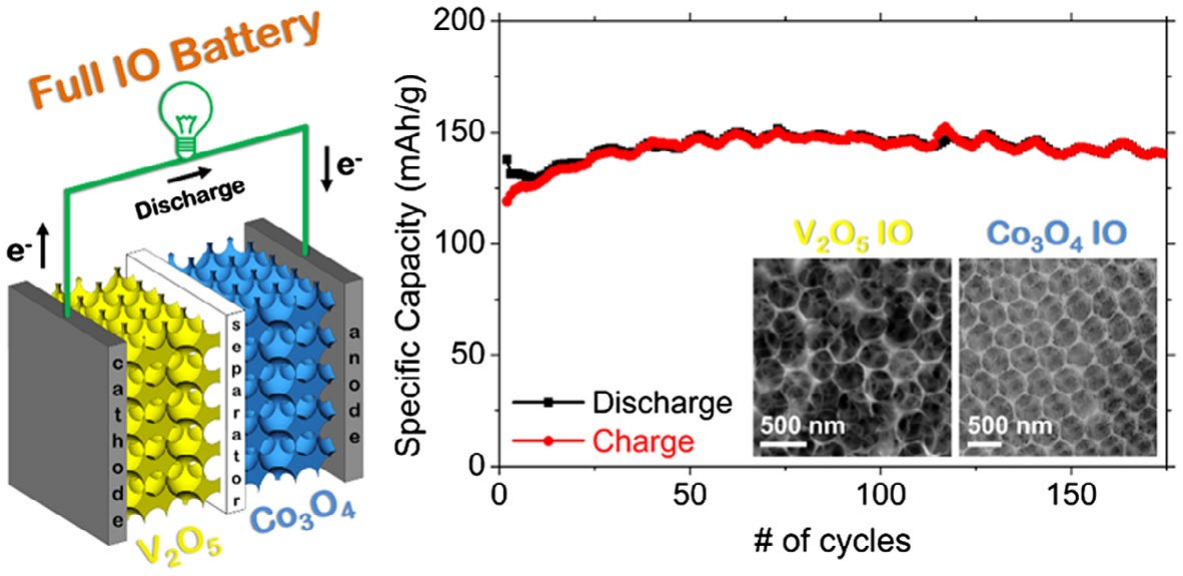
Schematic of a V_2_O_5_-Co_3_O_4_ inverse opal Li-ion battery cell. Efficient cycling without capacity fade is observed for over 175 cycles.

IO-based batteries and their capabilities were further extended in a recent study, where an all IO electrode Li-ion battery was demonstrated that contained an intercalation mode cathode with a conversion mode anode.[115] This non-traditional electrode mode pairing provided excellent capacity and cycle life, and effectively removed issues with initial capacity loss in the first cycles, typically of both types of electrodes. Electrochemically charged conversion mode Co_3_O_4_ IOs behaved as Li-ion anodes and the V_2_O_5_/Co_3_O_4_ cell cycled with superior performance compared to lithium batteries or half cells of each IO material on its own. Even under asymmetric slow discharge/fast charge tests, the all IO full Li-ion cell cycles without any capacity decay, as shown in Figure 7. These developments using 3D structured anodes and cathodes with new co-operative charge storage mechanisms provides alternatives for higher rate, high capacity, stable Li-ion batteries that can be extended to other pairs of materials with balanced modes of operation and capacity. As the benefits of IO structures for battery materials is now firmly established, the periodic 3D structure now provides possibilities for material metrology during electrochemical modification for new materials.

### 5.2. Properties of photonic crystals useful for electrochemistry and energy storage

Some phenomena and effects that occur in Li-ion battery and electrochemical energy storage devices during charging and discharging can be examined by monitoring the characteristics specific to inverse opal versions of those materials.[11] By imposing 2D or 3D periodic structure on any material,[116] it definitively becomes amenable to characterization via spectroscopy, diffraction and scattering (of light) from bandgap absorption (UV-vis), to scattering and diffraction (UV-vis-NIR), and PBG effects (vis-NIR) in ways bulks materials cannot.[7–9,16,17,117–121]

Outlined next are some key parameters of battery materials that are affected by certain electrochemical measurements – these are provided in the context of battery materials, but equally apply to systems where surface or bulk changes to physical properties of the material occur.

### 5.3. Intercalation, insertion, and alloying reactions during discharge and charge

Each of these mechanisms involves a reaction of some sort with lithium, or whatever cation is used (Mg, Na, Ca etc.) for post-Li-ion technologies.[122] For intercalation reactions, a modification of three (out of many) parameters that affect the definition of diffraction from IO or PhC materials, occur readily during discharging and charging. The first is the volumetric change that accompanies the uptake of Li into the crystal structure of the active material. This will either occur uniformly throughout the 3D porous network, or non-uniformly, and affects the periodicity.

If this occurs uniformly, it amounts to a thickening of the IO walls that comprise the network. Second, the intercalation of cations into anode or cathode materials markedly alter its conductivity, which in turn affects the dielectric constant as both are linked via $\eta \;\propto \sqrt{\varepsilon }$ and Drude theory. As the 3D PBG given by:

(1) $$\sqrt{8/\;3}D\sqrt{g\bar{\varepsilon_{s}}+\left(1-g\right)\bar{\varepsilon_{f}}}$$

which is a function of the dielectric constant, conductivity variations map onto diffraction spectra even without a change in material size or volume. Here, $\bar{\varepsilon_{s}}$ and $\bar{\varepsilon_{f}}$ are the permittivity values of the electrolyte in the pore of diameter *D*, and the IO wall material between these pores respectively, and *g* is the maximum packing factor for the face centred cubic (fcc) lattice, for example.

When both effects occur, strong modifications to periodicity and refractive index contrast provide a very sensitive way of following the effect of lithium insertion or alloying at different rates, over many cycles and for comparison of different materials fashioned into similar IOs. For conversion-mode battery reactions,[123,124] the formation of Li_2_O in tandem with electrochemical reduction of the metal oxide M_x_O_y_ to its metal M^0^ results in coverage of the electrode surface and pores with Li_2_O and other species such as carbonates, by a significant conversion and molar volume change for the oxide of the wall structure, which becomes metallic.

**Figure 8.**
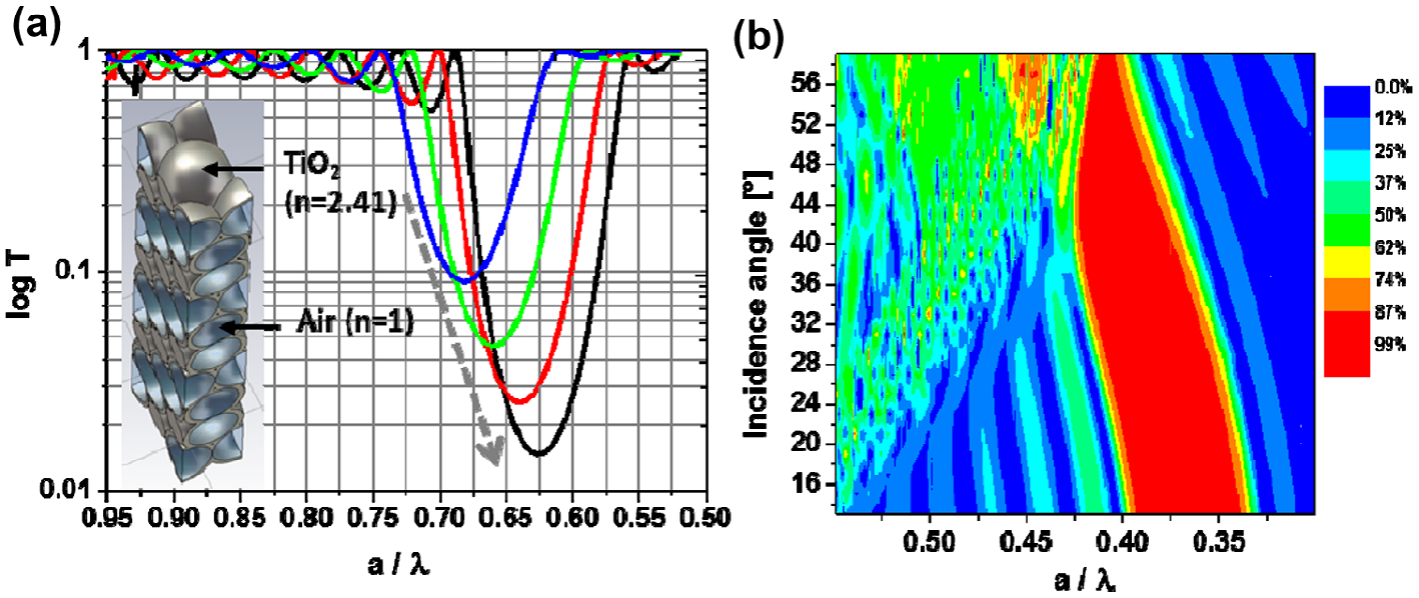
(a) Calculated transmission spectra of a 10 layer TiO_2_ inverse opal for various shell thicknesses. The grey arrow indicates the direction in which the shell thickness increases. (b) Contour plot of reflectance spectra of the TiO_2_ IO for different incidence angles. The oscillations outside stopgap correspond to the Fabry–Pérot fringes due to the finite sample geometry. Reproduced from [125] with permission from the Optical Society of America.

Lee et al. [125] like many others, have probed the angle-resolved reflectance spectra of inverse opals. For TiO_2_ IOs grown by repetitive vertical self-assembly and atomic layer deposition (ALD), they confirmed that the blue-shift of the stopband from the IO formed from the [111] planes of the parent opal follow the modified Bragg-Snell law:

(2) $$m\lambda_{m}=2d\sqrt{\eta_{eff}^{2}-\sin^{2}\emptyset_{1}}$$

where *η*_*eff*_ is the effective refractive index of the media and depends on the relative refractive indices of the material and medium within the pores. Since a feature of interest in battery materials is the variation in IO wall size during charging and discharging, it is useful to know if such a change is clearly observable from monitoring PBG spectra. Figure 8 shows simulated transmission spectra at fixed incident angle for a single TiO_2_ IO developed using a three-dimensional finite element model simulation of an inverse opal. In this case, the IO wall thickness was varied from 0.086*r* to 0.155*r*, where *r* is the radius of the pores. The transmissions dip shifts toward long wavelengths and increases in suppression as the wall thickness increases, which is consistent with the increase of average dielectric constant in the structure. This is precisely what can happen in an IO cathode or anode during discharging or charging respectively, and if the mole fraction of the new phase is known and swelling occurs uniformly throughout the IO, then the depth of discharge or state of charge can be correlated to the shift in transmission minima.

We note that analysing any electrochemical energy storage or conversion material with an IO structure in transmission mode is best achieved by avoiding higher incident angles, to minimize the contribution from diffuse scattering, and in some cases the propagation of ballistic photons – a clean reflectance maximum or PBG spectrum with angular sensitivity is also aided by smaller angles so that probe laser beam waists do not develop severe oblique profiles.

### 5.4. Metrology for electrochemical response of battery materials

While battery materials themselves are inherently disordered in their make up as an electrode, the use of the 3D or 2D IO structures outlined here helps to identify the material’s response to reversible cation intercalation, alloying or interactions (conversion mode, for example, or the effect of surface coatings), in real time or *ex situ*, non-destructively using the periodic (or changes in) *arrangement* of the material, rather than direct spectroscopic examination of the composition or crystallinity. But, it will become necessary to develop metrology techniques with improved accuracy to provide quantitative information, tailored to specific structures and processes. Tackling challenges of reduced dimensions and more complex structures (ordered or not) and multi-element materials with complex crystal structures, is challenging – metrology of electrochemical material behaviour will enable improved quality control and use of nanotechnology in industry.

The link between understanding of behaviour and performance can be incorporated into further materials processing steps so that the fundamental advances in cross-cutting analysis tools for materials electrochemistry can be supported with metrological optimization linked to materials performance.[126] Again, the photonic crystal or IO structure can be exploited to develop comparative metrology for some important battery materials.

For example, McDowell et al. [127] recently outlined how single nanowire (NW) battery cells, analysed using transmission electron microscopy (TEM), can be elegantly adapted to study the rate and alloying reactions during the lithiation and delithiation of a single Si NW, and factors that affect ultrafast charging have also been explored.[128] Here, it was important to understand how the crystal surface behaves under battery cycling conditions. For example, LiFePO_4_ can be significantly enhanced in terms of charging rate when coated with a thin carbon layer,[129] which acts as a lithium shuttle for ions around the particle surface to the crystalline facet that promotes faster and easiest ion intercalation. In all cases, the changes occur at electrode level or at single nanostructure level and there remains an open question as to how relevant single structure studies can be for large mixed composites, since mass loading density, local electric field and current densities can vary in random composites, and thus will any lithium reactions. However, these inform us about the nature of the interaction for rational changes to an important material, and so continue to drive advances in material composition for advanced battery materials. At the core of the importance of crystallinity and composition, is its relation to the solid state electrochemistry with respect to lithium interactions – the reader is referred to an excellent review by Goodenough [4] on the solid state chemistry of these concepts.^[4]^ There is a large body of evidence confirming the importance of surface orientation,[130] interfaces with solutions, and crystallographic orientation for materials in catalysis,[48,131–133] semiconductor micro-nanoelectronics (etching), charge storage, electrochemistry, sensors, etc., and the crystallographic orientations of crystalline materials have also become crucial for defining the important parameters of battery materials.[134–137]

Details on all-optical probing methods for electrochemical energy storage materials, where the vision for correlating the fundamental physics of opal photonic crystals, and many attributes of inverse opal structures to the nature of the electrochemical behaviour of materials arranged in such structures, can be found elsewhere.[39,138–141] Optical techniques are non-contact and fast, which makes them ideal for integrated process monitoring. In [39], considerations were given to optical diffraction on the scale of the IO, absorption on the scale of the nanomaterials that make up the walls of the structure, and PBG changes due to whatever electrochemical process occurs. However, subwavelength modifications to IO structures and nanomaterials are also critical in electrochemical energy storage, particularly for capacitor technologies and materials in batteries whose dielectric constant is not greatly modified, or whose volumetric expansion during lithium insertion or alloying is minimal. Of course, subwavelength changes to material size also occur in a plethora of other electrochemical systems.

**Figure 9.**
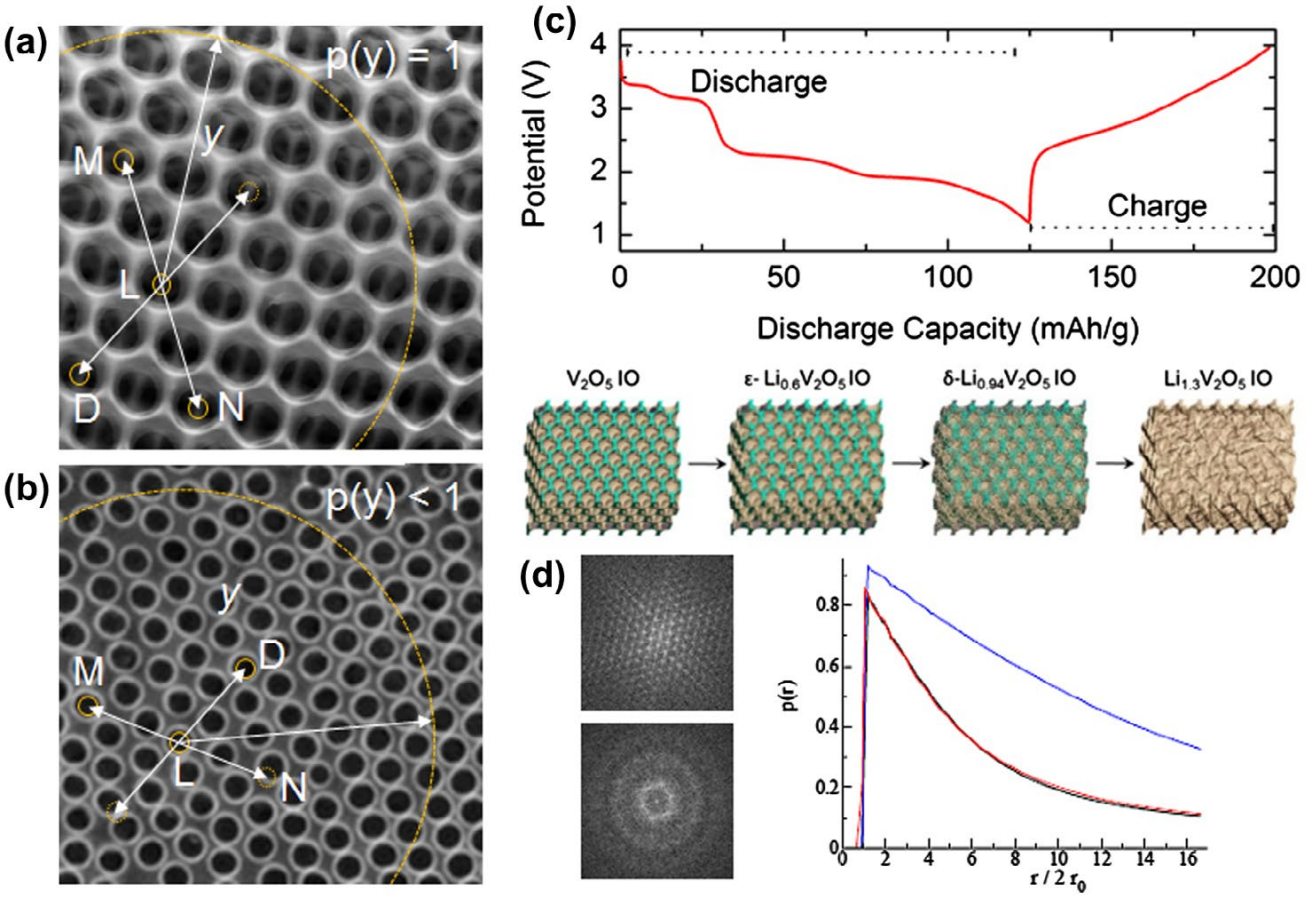
(a) V_2_O_5_ IO structure with high order. (b) a similar IO structure with a reduced degree or fcc ordering. The solid arrows indicate two spheres that are point-symmetric with respect to the centre void *L*. A *p*(*y*) = 1 implies perfect ordering and relative positing of void centres. In (a), there is always a perfectly positioned opposite partner void. When the distribution of opposite partners (which defines a deterioration in the lattice structure), *p*(*y*) < 1. The dashed arrow indicates a case in (b) for void *D*, where there is no opposite partner (similarly for *N* in this case). (c) Galvanostatic discharge-charge profile experimentally determined for a V_2_O_5_ IO cathode in a lithium battery. The schematic depicts the reduction in periodic, ordered structure that occurs during discharge, reproduced from [144] with permission from the American Chemical Society, 2015. (d) Example of the variation in *p*(*y*) as a function of the reduced *y* radius parameter, showing how the order reduction is measured from imagines of the IO top surface.

Alternative optical methods are also being developed currently, and can be adapted for electrochemical energy storage materials analysis. Using high resolution cameras with high frame rates, optical images of the electrode structure can be obtained during operation and analysed using computational methods to probe variation in the position and relative shift of the centroid of the ordered pores. A technique to quantify order by establishing the numbers of elements with a symmetrical partner has been developed for colloidal crystals of microspheres, and block copolymer arrays with periods of 10–40 nm.[142] It produces a single numerical value for the order, based on a given positional tolerance (Figure 9) and thus it may be possible to provide information about new materials using IOs that even PBG and optical scattering methods, as powerful as they are, cannot assess. Khunsin et al. showed that methods similar to pair correlation analysis and FT correlation allow comparative quantification of the degree or ordering with opal templates depending the assembly parameters; the same approach is feasible for inverse opals in 3D or 2D form, whereby the centroid of the void is displaced with respect to nearest neighbour and correlated pairs depending on the uniformity or non-uniformity of the IO wall thickening or thinning (reverse process) during discharging or charging. Fuller detail on analogies to bond orientation correlation and translation correlation are provided in [143,144], where the reader can see how the functions allow comparison of long range order and order in hexagonal arrangement in pristine vs. disordered states (after multiple cycles in a battery for example).

Briefly, we provide that definition reworked to accommodate the situation where a highly ordered hexagonal lattice of voids on the top of the IO electrodes is imaged at the beginning of a measurement, and at subsequent stages during electrochemical modification. Correlation-based statistical analysis from imaging provides a metrology to determine the way in which the periodic IO lattice changes during battery cycling, which can be compared between samples and materials or to optical spectra of the global 2D diffraction or 3D PBG changes. In light of the above requirements, Khunsin et al. introduced the function *p*(*y*) as a measure related to the sixfold symmetry of the in-plane hexagonal lattice of an opal – we redefine it for an inverse opal here. The measure of IO regularity is based on the probability of finding an opposite partner sphere (see Figure 9). The regularity measure *p*(*y*) works as follows: for two randomly chosen IO voids *L* and *M*, *p*(*y*) is defined as the probability of finding a third void *N* such that *L*, *M*, and *N* are collinear and that the distance between *L* and *M* is identical to that between *L* and *N* (within the tolerance defined by the image resolution) as depicted in Figure 9.

The local regularity measure at a chosen sphere *A* can be defined as *p*_*L*_(*y*) = *O*_*L*_(*y*)/ *N*_*L*_(*y*), where *O*_*L*_(*y*) is the number of voids within a circle of radius *y* around the void *L* that have a symmetric opposite and identical partner void and *N*_*L*_(*y*) is the total number of voids within this circle not counting the central void. Thus the measure is defined as:

(3) $$p\left(r\right)=\frac{\sum_{L}N_{L}\left(y\right)p_{L}\left(y\right)}{\sum_{L}N_{L}\left(y\right)}$$

For a perfectly ordered hexagonal lattice with outermost (111), *p*(*y*) = 1 for all *y* values. This value of course decreases as disorder is introduced to the lattice caused by differential thickening of the IO walls and displacement of the centroids of the voids. Of particular interest is that the symmetric opposite neighbouring void approach allows for measurement of crystallographic texture changes in a lattice arrangement, and as the starting lattice can be defined, relative changes are measureable without a perfect starting lattice.

There are possibilities to adapt both concepts of imposing a 3D photonic crystal structure on the material, and by determining the relative change with each pore that contributed to the disorder by tandem optical spectroscopy and image analysis.[145] By patterning of single crystals of a material into holey arrays or IOs to delineate certain facets around a void (the rim being the facets of the crystal), information on the movement or changes of the crystal facet during an electrochemical measurement can be tracked via FT analysis of surface images. Using the method outlined above, the degree of disorder normally captured on the whole by PBG monitoring, can be ascribed to the shift or change of each individual hole in the array. Based on the intensity and equality of harmonics in the transform, FT image analysis is more sensitive to the grey level of the matrix between the particles, and since the relationship between FT features and crystallographic order is very well understood, it provides a reference for use of any matching elements method for image analysis to observe the response of the material around each pore in the array.

Lastly, the slow photon effect pioneered by Ozin and co-workers [62,67,68,78] that incorporates variations in effective group velocity and photonic band edges of IO structures can also be adapted for battery material with an imposed 3D IO structure.

#### 5.5. 2D patterned arrangements of battery materials

Arranging a material in a patterned photonic crystal-like arrangement as a 2D planar array or structure, allows the ‘photonic crystal’ structure to behave similar to a diffraction grating. Clearly, such a thin structure with any defined and dominant ordered porosity that promotes optical diffraction or scattering, will severely limit the gravimetric energy density (low mass loading) of the electrode. The purpose is not to benchmark performance (cycle life, capacity, energy density, etc.), but to impose a certain periodic architecture on any material and characterize the changes to this ordered structure during electrochemical modification, to gauge any specific response to lithium reactions during an electrochemical measurement.

**Figure 10.**
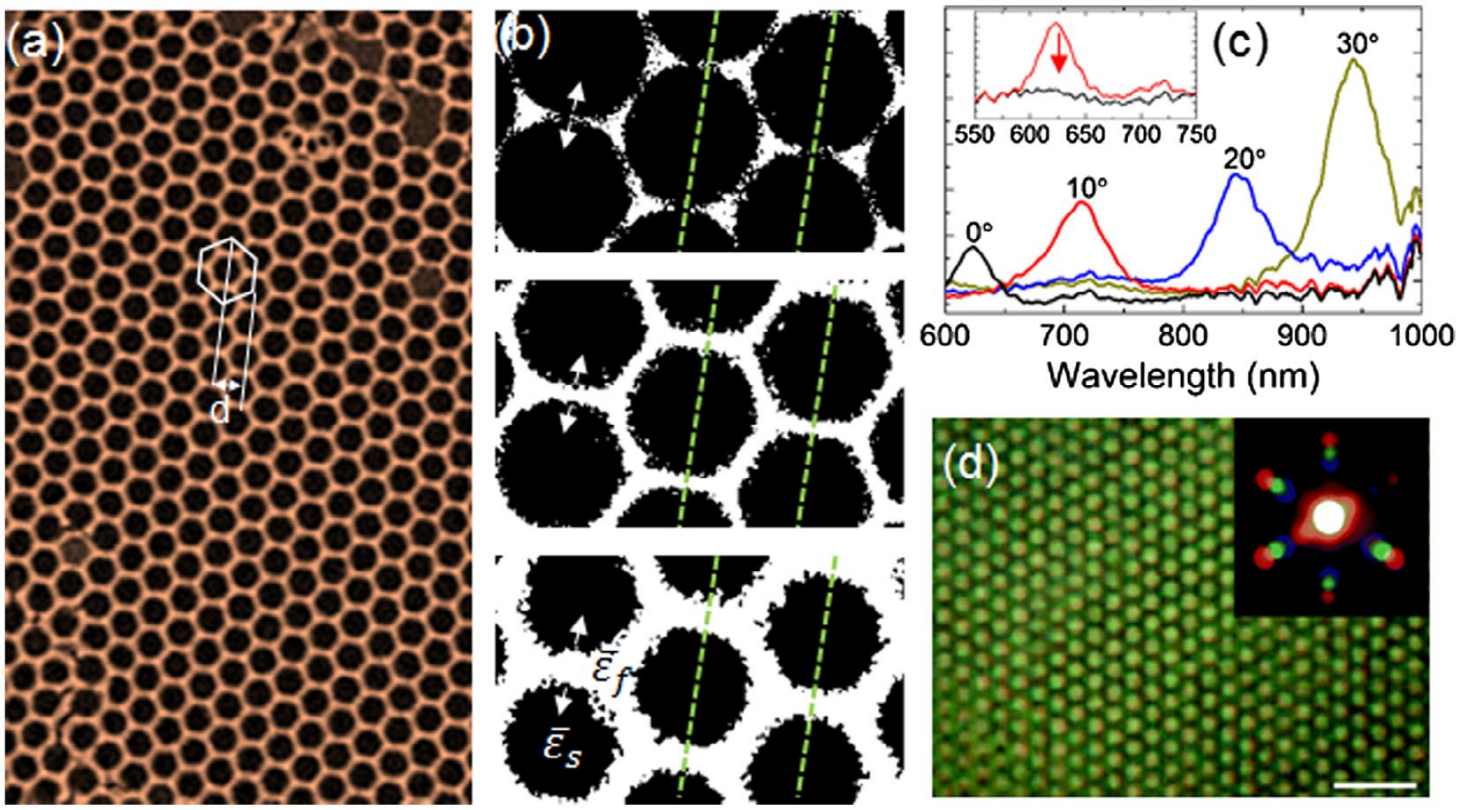
(a) 2D IO of SnO_2_ formed from Sn(II) acetate directly on silicon. The effective grating spacing *d* is defined as the half-period of the trigonal lattice formed by the IO. In (b), the variation of the IO walls as a function of electrochemically induced isotropic swelling is diagrammatically represented. The 2D planar grating period is overlaid. Here, the ratio of dielectric constants changes. (c) Angle resolved reflectance spectra for an opal 2D diffraction grating and (inset) the suppression of diffraction following the onset of lattice disorder. (d) An inverse opal from azo-benzene that projects a diffraction pattern from white light with a wavelength-dependent dispersion of diffraction maxima. These features are sensitive to short range order modification.

In using a 2D IO structure, there are some benefits in this regard. First, the 2D structure removes any issues with interconnectivity within, for example, a 3D IO structure, i.e. there is just one ‘layer’ of material and concerns about material interconnectivity and electronic transport are assessable by checking the IO walls in the 2D arrangement using microscopy – cracks are most obvious in this structure, and internal cracking is not always easy to find within a 3D IO. An example of a high quality 2D IO of SnO_2_ and electron microscopy of the NP network is shown in Figure 10.

Second, 2D IOs with typical primary pore widths of 200 nm to 10 μm easily accommodate complete electrolyte soakage for electrochromic, batteries, supercapacitors, aqueous or aprotic electrochemistry, water splitting or solar to fuel applications to name just a few. This ensures that diffraction analysis of 2D IOs during electrochemical modification can be related to changes in the material volume, whether periodic or aperiodic.

Third, the majority of all electrochemical reactions, including energy storage and conversion, takes place at the surface, or begins at the surface, and so efficient electron transfer is required at the material–solution interface. While the electrolyte interface is defined for the open-worked 2D IO, the interconnectivity of a conductive material is also important. As seen in Figure 10, even a granular, nanoparticle-containing IO wall structure of a 2D IO is well defined and since electrical transport is a function of the material conductivity and conduction path through the opal network, efficient electrochemical reactions can be assessed by treating the electrode as a planar diffraction grating. This cannot be achieved using 3D IOs or indeed the vast majority of randomly arranged particle composites or agglomerates. A 2D IO that behaves as a diffraction grating provide a response in the visible spectrum, even when the voids are of a dimension that cause a photonic band gap in the infrared if fashioned as a 3D IO. The fidelity and wavelength of the optical diffraction pattern is sensitively linked to the modification of the grating’s groove spacing, or 2D IO arrangement during an electrochemical measurement.

Additionally, 2D IOs fashioned from colloidal opal templates of polymer spheres have very good adhesion to the substrates, electrode surface or current collector – extensive cracking due to capillary forces from electrolyte soakage, and crack formation and propagation during drying is far less likely in a 2D IO-type structure compared to a 3D structure.[146] This minimizes complexity in analysis of optical diffraction spectra when there are variations in periodicity (grating period) from material swelling, or from conductivity/dielectric constant modification due to the electrochemical process that occurs at or within the material (the contrast assumes knowledge of the refractive index of whatever electrolyte is within the pores).

For a 2D IO where there is a periodic variation in refractive index, we can treat the system much like the parent opal (but with a redefinition of the index contrast based on the inverse structure) and geometrically define the comparison to a 2D diffraction grating. In this sense, identification of a fixed diffracted wavelength at a particular angle can be monitored during the electrochemical experiment, yielding an *in operando* assessment of structure, surface, conductivity and other processes that occur in battery and related materials (shown in Figure 10).

The planar dielectric grating equation provides the theoretical dispersion of diffracted light from a 2D artificial opal [138,147] and can be reworked to apply to the inverse opal structure. Discrete angles (*β*) for a given effective groove spacing (*d*), depicted in Figure 10(a) and 10(c), are defined so that constructive interference occurs between diffracted light, $\lambda =d\left[\sin\left(\alpha \right)+\sin\left(\beta +x\right)\right]$, where *d*, in the case of a monolayer 2D IO, corresponds to the half period of the trigonal lattice $\frac{\sqrt{3}}{2}$ *D* where *D* is the void diameter, *α* is the angle of incidence, *β* the angle of diffraction, and *x* is a free constant commonly used in data fitting, and is associated with the discrepancy in diffraction energy caused by the assumption in similarity between the inverse material arrangement and that of a planar groove in a diffraction grating. Here we note this effect in the change in the diameter of the void and the thickening of the IO walls, even though the periodicity remains unchanged.[148] Accounting for the variation in refractive index by changes in fill fraction (ratio of electrolyte filled pore to inorganic material walls, may be achieved following *η*_avg_ = [ϕ *η*_solid_ + (1 – ϕ) *η*_void_], where ϕ is the fill factor of the solid material. Monitoring any reduction in the full width at half maximum (FWHM) also provides information on the ordering and degree of modification to the 2D order caused by electrochemical reactions and changes to the material.

While 3D IOs provide a sensitivity to variations in periodicity via the photonic bandgap, 2D monolayer IOs with thickness far below an equivalent Bragg attenuation length thus cannot show any 3D effects in the visible range. Unlike 2D opals made from large spheres whose diameter is similar to the wavelength of the light, 2D IOs do not provide any strong Fabry–Pérot resonances, which are negligible compared to scattering or diffraction contributions. In this case, at a constant angle, we may monitor the change in intensity and FWHM, or bandwidth, of the diffraction peak. As shown in Figure 10(b), a fixed widening of the material size will affect the diffraction wavelength, and the dispersion of the ratio of the bandwidth to the resonant wavelength of the diffraction peak. The specific measurements are powerful in assessing how uniform the volumetric change in material is, as *lateral* changes in the periodicity and index contrast of the 2D IO, and thus the grating period and the diffracted light.

## 6. Conclusions and outlook

Some recent advances and concepts in IO structures and their application were provided with a focus on their use as catalysts, catalyst support materials, as electrodes for batteries, water splitting photoanodes, solar to fuel conversion and electrochromics, and as photonic photocatalysts and photoelectrocatalysts. 3D periodically porous materials with unique optical properties that define the light–matter interactions that prompted much of the findings referenced here, have been briefly reviewed for electrochemical energy conversion systems as well as photocatalytic systems. Strategies for using 2D as well as 3D structures, ordered macroporous materials such as IOs were also compounded with recent developments in plasmonic–photonic coupling methods in metal nanoparticle-infiltrated semiconducting metal oxide inverse opals for enhanced photoelectrochemistry.

A combination of the open-interconnected network structure, with walls that are often made form nanocrystals or nanoparticles of the material, provide very good gas and liquid access, and a high surface area for a variety of catalytic, chemical or electrochemical interactions and reactions. The optical properties of photonic crystals have shown to be very useful in providing more efficient photoelectrochemical electrodes, photo- and electro-catalysts, and also Li-ion battery materials. Structured materials, with order or without, provide general catalytic enhancement mechanisms and for some systems that do not require absolute perfection in the IO lattice, easy synthesis to enable a diverse variety of materials with opportunities for easy dopant incorporation, composites and complex multi-component/heterojunction and multifunctional materials. If one is careful with the choice of structure, porosity and arrangement based on the required use, there are synergistic effects from the IO optics and the material (electro)chemistry that can clearly be exploited.

Tunable optical properties can enhance light–matter interaction for photocatalytic and photoelectrochemical performance but there are a myriad of requirements for good IO creation (such as defects that cause Mie or Rayleigh scattering as one example) that must be met in order to exploit their optical properties fully – we acknowledge the difficulty in preparing high quality IOs in large amounts, and without subsequent damage when exposed to subsequent infiltration steps for NPs attachment (not all, but some have highly reducing solutions and solvents that promote solubility). Many of the available results and reported efficiencies in IO-based photoelectrochemical systems may be a lower bound. As has been detailed with 3D materials for batteries, photoelectrochemical and catalytic systems are efficient if the surfaces of the materials are active, and thus the possibility for side reactions needs to be considered. In an inverse opal, electronic conductivity, mobility, grain boundary resistance, ohmic drops, interfacial contaminants, etc., all affect charge transport in a material that is difficult to make without sub-wavelength defects that can suppress the very optical properties that are needed. Thus, there is potentially a trade-off in the search for scalable, cheap and easy ways to fabricate high quality IOs, with the efficiency gains in solar to fuel production – some more expensive but superior infiltration and coating methods may provide a better test of some of the most efficient systems reported to date that rely on photonic crystal properties for their improvement. Compared to batteries where some enhancement is provided by porosity, the synergistic electro-optical effects rely on a well-defined PBG from homo- or heterogeneous single or multilayer photonic crystal structures, plasmonic–photonic coupling, slow photons, resonant amplification in a 3D PhC cavity and other effects that really rely on very high quality material infiltration methods. It remains to be seen if the photonic crystal quality of optoelectronics can readily provide much better and also more stable PEC systems.

## Disclosure statement

No potential conflict of interest was reported by the authors.

## Funding

GC acknowledges a National University of Ireland Fellowship in the Sciences. This work was also supported by Science Foundation Ireland (SFI) through an SFI Technology Innovation and Development Award [contract number 13/TIDA/E2761]. This publication has also emanated from research supported in part by a research grant from SFI [grant number 14/IA/2581].
